# Neuroligins and Neuropathic Pain: Insights into Synaptic Plasticity and Pain Transmission

**DOI:** 10.3390/biology15141180

**Published:** 2026-07-17

**Authors:** Mario García-Domínguez

**Affiliations:** Facultad de Educación, Universidad Alfonso X El Sabio (UAX), Avenida de la Universidad, 1, Villanueva de la Cañada, 28691 Madrid, Spain; marigado@uax.es

**Keywords:** neuropathic pain, neuroligins, synaptic plasticity, central sensitization, neuroinflammation, excitatory/inhibitory balance

## Abstract

Neuropathic pain is a chronic condition resulting from injury or dysfunction of the nervous system, characterized by hyperalgesia, allodynia, and persistent alterations in sensory perception. Neuroligins, a family of postsynaptic adhesion proteins essential for synapse formation and maturation, have emerged as critical regulators of neuronal plasticity and nociceptive circuit excitability. Recent evidence suggests that dysregulation of neuroligin expression and function can modulate synaptic transmission in pain pathways, contributing to the onset and maintenance of neuropathic pain. This review highlights some findings on the role of neuroligins in neuropathic pain pathophysiology, underscoring molecular mechanisms, interactions with glutamatergic and GABAergic receptors, and their potential as innovative therapeutic targets for chronic pain management.

## Simple Summary

Neuropathic pain is a condition caused by injury or dysfunction of the nervous system, characterized by hyperalgesia, allodynia, and sustained alterations in sensory processing. Neuroligins are postsynaptic adhesion proteins essential for synapse formation and maturation. Emerging evidence indicates that dysregulation of neuroligin function can alter synaptic transmission within pain pathways, thus contributing to the development and maintenance of neuropathic pain. Overall, this review highlights the role of neuroligins in the pathophysiology of neuropathic pain, emphasizing their interactions with glutamatergic and GABAergic systems and their potential as novel therapeutic targets for chronic pain management.

## 1. Introduction

Neuropathic pain is a chronic and disabling condition that arises from damage to, or dysfunction within, the somatosensory nervous system, involving peripheral and central pathways [1]. Unlike nociceptive pain, which represents a physiological response to tissue injury, neuropathic pain is driven by distinct pathophysiological mechanisms, including increased neuronal excitability, peripheral and central sensitization, disturbances in neuroimmune signaling, and impaired synaptic plasticity [2,3,4]. Clinically, this condition is characterized by symptoms such as spontaneous pain, burning or stabbing sensations, electric shock-like attacks, allodynia, hyperalgesia, paresthesia, and, in several cases, sensory deficits [5]. These symptoms typically persist well beyond the resolution of the initial insult, indicating enduring alterations in neural function rather than ongoing peripheral damage [6]. Neuropathic pain is usually linked to a variety of clinical disorders, including diabetic peripheral neuropathy (DPN) [7], chemotherapy-induced peripheral neuropathy (CIPN) [8], postherpetic neuralgia [9], multiple sclerosis [10], and spinal cord injury [11]. As a result, it constitutes a major source of long-term disability, emotional distress, and diminished quality of life across affected populations, posing a considerable burden on both patients and healthcare systems [12,13].

At the mechanistic level, neuropathic pain is increasingly conceptualized as a disorder of maladaptive neuroplasticity. This maladaptive process entails coordinated changes at both peripheral and central levels of the nervous system. At the peripheral level, sensitization results from increased excitability of primary afferent neurons, driven by changes in ion channel expression and intracellular signaling cascades, which give rise to spontaneous ectopic activity and exaggerated responses to sensory input [14,15]. Within central neural circuits, sustained nociceptive input drives central sensitization within the spinal dorsal horn and supraspinal circuits, defined by increased synaptic efficacy, reduced activation thresholds, elevated receptive fields, and reduced inhibitory controls [16]. These changes are mediated by enhanced glutamatergic transmission, particularly via upregulated NMDA and AMPA receptor activity, alongside concurrent dysfunction of GABAergic and glycinergic inhibitory signaling [17,18].

Synaptic plasticity provides a useful framework for understanding how these neurobiological changes become persistent. Synaptic plasticity covers functional and structural changes at synapses, including long-term potentiation (LTP), long-term depression (LTD), dendritic spine remodeling, and alterations in synaptic protein composition [18,19]. Synaptic plasticity in pain circuits is very dynamic and regulated by numerous molecular factors, among which synaptic adhesion molecules have emerged as key modulators [20].

Synaptic adhesion molecules are essential for the formation, specification, and stabilization of synaptic contacts. Among these, neuroligins have attracted considerable attention due to their essential role in organizing synaptic architecture and regulating the neurotransmission. Neuroligins are postsynaptic cell adhesion proteins that engage in trans-synaptic interactions with presynaptic neurexins, forming a molecular bridge that aligns pre- and postsynaptic structures [21,22]. The neuroligin family includes several isoforms, each with different localization patterns and functional roles within the nervous system. Neuroligin-1 (NLGN1) is mainly localized at excitatory glutamatergic synapses, where it interacts with scaffolding proteins such as PSD-95 to regulate the clustering of AMPA and NMDA receptors [23]. Neuroligin-2 (NLGN2), in contrast, is usually linked to inhibitory GABAergic synapses and interacts with gephyrin to stabilize GABA_A_ receptors [24]. Neuroligin-3 (NLGN3) exhibits a more versatile distribution, being present at both excitatory and inhibitory synapses [25], while neuroligin-4 (NLGN4) has been implicated in specialized synaptic functions [26].

In addition to their role in synaptic organization, neuroligins are increasingly recognized as modulators of intracellular signaling pathways that regulate synaptic plasticity. Through interactions with scaffolding proteins and signaling complexes, neuroligins influence receptor trafficking, synaptic vesicle release probability, and cytoskeletal dynamics [27,28,29]. These processes are crucial for the induction and maintenance of LTP and LTD, which are thought to underlie central sensitization in neuropathic pain [30,31]. Moreover, neuroligins may interact with specific neuromodulatory systems and neuroimmune signaling pathways, thereby integrating numerous regulatory mechanisms within pain circuits [32,33,34].

Another important aspect of neuroligin function in neuropathic pain is their potential involvement in structural plasticity. Changes in dendritic spine density and morphology have been documented in pain-processing regions following nerve injury, reflecting alterations in synaptic connectivity [35]. Given their role in synapse stabilization, neuroligins are well positioned to participate in these structural adaptations [36,37]. These structural modifications further strengthen functional changes in synaptic transmission, creating a self-sustaining cycle of neural hyperexcitability.

Importantly, the growing recognition of neuroligins in neuropathic pain offers promising new directions for therapeutic intervention. Targeting synaptic adhesion molecules provides an innovative approach to modulating neural circuit function at a fundamental level, with the potential to restore excitatory–inhibitory balance. Nevertheless, developing neuroligin-based therapies introduces considerable challenges, such as achieving isoform specificity, ensuring precise spatial targeting, and minimizing unintended effects on normal synaptic activity. Progress in molecular biology, gene-editing technologies, and targeted drug delivery systems could help address these challenges and support the translation of basic research into clinical applications.

Accordingly, this review provides a comprehensive overview of the role of neuroligins in neuropathic pain. It synthesizes current evidence regarding their molecular functions, interactions with neurotransmitter systems, and contributions to synaptic plasticity. The objective is to clarify the mechanisms by which neuroligins influence pain transmission and persistence while evaluating their potential as therapeutic targets. By integrating findings from molecular, cellular, and translational studies, this review aims to advance understanding of neuropathic pain as a disorder of synaptic dysfunction and to identify promising directions for future therapeutic development.

## 2. Neuroligins: Structure and Biological Functions

The neuroligin family is composed of a group of postsynaptic cell-adhesion proteins within the immunoglobulin superfamily that are essential for the initiation, maturation, and refinement of synaptic contacts in the mammalian CNS [21,22]. These proteins are not only adhesion molecules but also key molecular organizers of synaptic nanoarchitecture, coordinating the spatial and functional alignment of presynaptic release machinery with postsynaptic receptor fields [38].

Structurally, neuroligins are type I single-pass transmembrane glycoproteins with a modular organization that includes an extracellular ligand-binding domain, a single hydrophobic transmembrane α-helix, and a short but functionally dense intracellular C-terminal tail [39]. This domain architecture enables neuroligins to function as bidirectional trans-synaptic scaffolds, coupling presynaptic neurexins to postsynaptic scaffolding complexes and thereby contributing to the molecular specification of excitatory and inhibitory synaptic identity.

### 2.1. Neuroligin Family Overview

At the structural level, neuroligins display a conserved tripartite organization consisting of (1) an extracellular cholinesterase-like (ChE-like) domain, (2) a single-pass α-helical transmembrane segment, and (3) an intracellular C-terminal domain enriched in short linear interaction motifs [40]. Despite homology to acetylcholinesterases, neuroligins are enzymatically inactive due to degeneration of the catalytic triad (Ser–His–Glu), with residue substitutions abolishing hydrolytic activity while preserving the α/β hydrolase scaffold [41]. This exaptation reflects an evolutionary shift from enzymatic catalysis to high-affinity molecular recognition, repurposing the fold as a stable synaptic adhesion platform. Conserved cysteines and disulfide networks further stabilize the extracellular domain, ensuring structural integrity under synaptic mechanical constraints [42,43].

**Figure 1:**
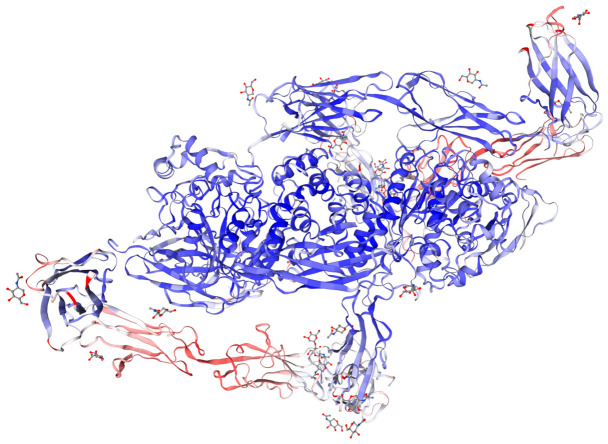
Tridimensional structure of human NLGN1. Image generated using Expasy software 3.0.

The extracellular ChE-like domain is the largest and most structurally complex region of neuroligins. It adopts a canonical α/β hydrolase fold comprising a central β-sheet flanked by α-helices (Figure 1) [44], stabilized by multiple disulfide bridges essential for extracellular stability [45]. Projecting into the synaptic cleft, it mediates presynaptic neurexin binding via conserved hydrophobic and complementary charged surface patches, enabling high-affinity, Ca^2+^-dependent interactions [46]. Structural robustness is reinforced by glycosylation and rigid loop architecture, which reduce proteolytic susceptibility and dampen activity-induced conformational fluctuations [47].

A defining structural feature of neuroligins is obligate homodimerization mediated by a conserved interface within the extracellular domain [48]. Dimer formation involves β-sheet extension, hydrophobic packing, and backbone hydrogen bonding, resulting in a stable head-to-head configuration [49]. This quaternary assembly is required for neurexin engagement by generating a bivalent binding platform that increases avidity through cooperative interactions [50]. Each neuroligin dimer can simultaneously bind multiple neurexin molecules in a Ca^2+^-dependent manner, with Ca^2+^ coordinating LNS (laminin–neurexin–sex hormone-binding globulin) domains and stabilizing the adhesion complex [51]. This multivalent architecture enhances synaptic specificity and reduces dissociation under mechanical stress.

The extracellular domain is extensively N-glycosylated at conserved asparagine residues located in solvent-exposed loops [52]. These glycans facilitate co-translational folding, prevent aggregation, regulate trafficking efficiency, and stabilize surface expression at the postsynaptic membrane [53,54]. In addition, glycosylation sterically modulates protein–protein interfaces, thus fine-tuning neuroligin–neurexin binding kinetics and shaping synapse-specific affinity profiles [55]. Alternative splicing at canonical sites (notably inserts A and B) introduces short peptide cassettes into extracellular loops, altering charge distribution and steric accessibility [56]. These isoform-specific modifications regulate selectivity for α- and β-neurexins and contribute to synaptic diversity and circuit-specific connectivity [57].

The transmembrane domain consists of a single-pass α-helix enriched in hydrophobic residues, ensuring stable insertion into the lipid bilayer [49]. Beyond structural anchoring, it contributes to lateral membrane organization by promoting partitioning into cholesterol-rich microdomains and postsynaptic density-associated nanoclusters [42]. Helix–lipid interactions shape membrane thickness matching, rotational dynamics, and interactions with other membrane proteins. In addition, this segment acts as an allosteric conduit, delivering extracellular binding-induced constraints to intracellular scaffolding interfaces and enabling mechanotransductive signaling across the synaptic membrane [58,59].

The intracellular C-terminal domain, although relatively short, is enriched in short linear motifs that mediate interactions with postsynaptic scaffolding machinery [60]. A canonical class I PDZ-binding motif at the extreme C-terminus (typically ES/TXV) enables direct binding to PDZ domains of major MAGUK scaffold proteins, including PSD-95 and SAP102 [60,61]. These interactions organize glutamatergic synapses by clustering AMPA and NMDA receptors and stabilizing receptor nanodomains within the postsynaptic density [62]. At inhibitory synapses, neuroligins interact indirectly with gephyrin-based scaffolds via adaptor proteins, thereby regulating GABA_A_ receptor clustering and inhibitory synaptic strength [63]. The intracellular domain thus plays an important role in synaptic subtype specification through differential scaffold recruitment.

Post-translational modifications provide dynamic regulation of neuroligin function. Phosphorylation of serine and threonine residues modulates PDZ-binding affinity, regulates trafficking between intracellular compartments and the plasma membrane, and influences activity-dependent synaptic remodeling [38,55]. Kinase-dependent signaling cascades can therefore adjust neuroligin surface expression, contributing to synaptic plasticity mechanisms such as LTP and LTD [64]. Ubiquitination at lysine residues further regulates neuroligin turnover via proteasomal degradation or endosomal–lysosomal pathways, enabling controlled removal of adhesion complexes during synaptic pruning or activity-dependent refinement [65].

At the quaternary and supramolecular level, neuroligins function as components of trans-synaptic adhesion complexes spanning the synaptic cleft. These complexes couple presynaptic active zones enriched in VGCCs and the synaptic vesicle release machinery to postsynaptic receptor nanoclusters [66]. The neuroligin–neurexin interaction is stabilized by Ca^2+^ ions, which coordinate binding loops within neurexin LNS domains and promote high-affinity docking. This molecular bridge ensures nanoscale alignment between neurotransmitter release sites and receptor fields, optimizing synaptic transmission fidelity, reducing diffusion loss, and increasing temporal precision of synaptic currents [67]. The resulting nanocolumn architecture is increasingly recognized as a key determinant of synaptic strength and neuronal information processing [68].

Isoform-specific diversification within the neuroligin family provides structural and functional specialization across synaptic subtypes. The following table (Table 1) summarizes the detailed characteristics of each neuroligin subtype.

### 2.2. Biological Functions of the Neuroligin Family

#### 2.2.1. Neuroligins in Synapse Formation and Activity-Dependent Maturation

During early neurodevelopment, neuroligins play a central role in the transition from transient axon–dendrite contacts to fully functional synapses [30]. This process, synaptogenesis, is not a passive stabilization event but an active and tightly regulated molecular program in which neuroligins act as key organizers. Upon binding presynaptic neurexins, neuroligins promote the recruitment of presynaptic active zone proteins, including components involved in synaptic vesicle docking and neurotransmitter release [91]. In parallel, postsynaptic neuroligins recruit scaffolding proteins such as PSD-95 at excitatory synapses and gephyrin at inhibitory synapses, establishing a molecular framework for receptor clustering and synaptic specialization [92,93]. This coordinated bidirectional organization ensures precise alignment of synaptic elements and supports efficient neurotransmission across the synaptic cleft.

As synapses mature, neuroligins continue to regulate structural refinement and functional stabilization through activity-dependent mechanisms. Synaptic activity modulates neuroligin trafficking and conformational state, thereby altering surface expression and interactions with neurexins in an activity-dependent manner [94,95]. This regulation is closely coupled to dendritic spine morphogenesis, in which immature filopodia-like protrusions develop into stable mushroom-shaped spines capable of sustaining long-term synaptic transmission [96]. Neuroligins contribute to this transition by modulating cytoskeletal dynamics through intracellular signaling pathways such as Rho family GTPases [36]. Accordingly, neuroligins function not only as structural organizers but also as key regulators of synaptic plasticity, integrating extracellular adhesion cues with intracellular remodeling processes underlying learning and memory formation [27,97].

#### 2.2.2. Regulation of Excitatory Synapses and Glutamatergic Transmission

**Figure 2:**
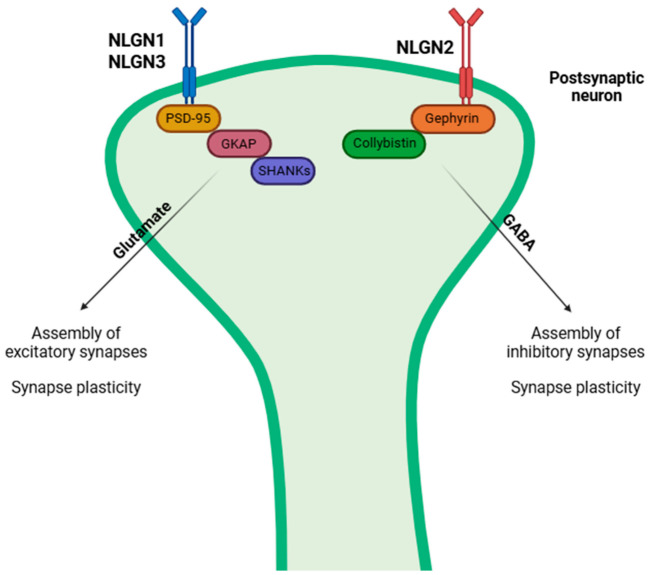
This figure illustrates the role of neuroligin-mediated postsynaptic scaffolding complexes in the assembly and functional specialization of excitatory and inhibitory synapses. NLGN1 and NLGN3 are predominantly associated with glutamatergic excitatory synapses, where they interact with PSD-95, GKAP, and SHANKs to regulate synaptic maturation. However, NLGN2 localizes mainly to GABAergic inhibitory synapses, where it recruits the scaffold proteins gephyrin and collybistin to promote inhibitory synapse assembly and stabilization. Through these molecular interactions, neuroligins coordinate the balance between excitatory and inhibitory neurotransmission, thus contributing to synapse formation, maintenance, and activity-dependent synaptic plasticity. Abbreviations: NLGN1 (neuroligin 1), NLGN2 (neuroligin 2), NLGN3 (neuroligin 3), PSD-95 (postsynaptic Density Protein-95), GKAP (guanylate kinase-associated protein), SHANK (SH3 and multiple ankyrin repeat domains protein), and GABA (γ-aminobutyric acid).

A key aspect of neuroligin function is their role in the formation and regulation of excitatory glutamatergic synapses. NLGN1 is the most extensively studied isoform in this context and is principally localized at excitatory synapses, where it contributes to postsynaptic density organization and receptor composition [98]. At these sites, NLGN1 directly interacts with PSD-95, a key scaffolding protein that anchors ionotropic glutamate receptors, including AMPA and NMDA receptors, at the postsynaptic membrane [60]. This interaction supports the structural and functional maturation of excitatory synapses, enabling efficient synaptic transmission (Figure 2) [99].

The regulation of AMPA and NMDA receptor localization by neuroligins is particularly important for synaptic plasticity mechanisms such as long-term potentiation (LTP), which underlies learning and memory [63]. AMPA receptors mediate excitatory postsynaptic currents [100], whereas NMDA receptors function as coincidence detectors due to their voltage-dependent Mg^2+^ block and Ca^2+^ permeability, triggering intracellular signaling cascades that promote synaptic strengthening [101]. By stabilizing these receptors at synaptic sites, neuroligins regulate neuronal excitability and enable activity-dependent synaptic plasticity.

Neuroligin-mediated regulation of glutamatergic transmission ensures precise synaptic integration and faithful processing of excitatory inputs [100]. Disruption of this system can lead to altered synaptic strength, impaired plasticity, and aberrant excitatory signaling, all of which are commonly associated with neurodevelopmental and neuropsychiatric disorders [102].

#### 2.2.3. Regulation of Inhibitory Synapses and GABAergic Transmission

In contrast to excitatory specialization, NLGN2 is predominantly localized at inhibitory synapses, where it plays a key role in orchestrating GABAergic transmission [103]. These synapses use GABA as their principal inhibitory neurotransmitter, and their proper function is essential for maintaining circuit stability and preventing excessive excitation [104]. NLGN2 associates with gephyrin, a scaffolding protein that mediates clustering of GABA_A_ receptors at the postsynaptic membrane [24]. This organization enables inhibitory synapses to generate hyperpolarizing currents that reduce neuronal firing probability.

Beyond receptor clustering, NLGN2 contributes to the regulation of network oscillations, neuronal synchrony, and excitation–inhibition (E/I) balance across neural circuits [105,106,107]. Inhibitory transmission mediated by NLGN2 constrains excitatory signaling in both spatial and temporal domains, preventing pathological hyperexcitability. Impairment of NLGN2 function destabilizes inhibitory synapses, leading to reduced GABAergic signaling and a shift toward excessive network excitation [108]. This imbalance is implicated in disorders like epilepsy, anxiety disorders, and autism spectrum disorder (ASD), in which inhibitory control is disrupted [109,110,111].

#### 2.2.4. Integration with Neuromodulatory Systems and Intracellular Signaling Pathways

Beyond their role in organizing glutamatergic and inhibitory GABAergic/glycinergic synapses, neuroligins have also been implicated in the modulation of cholinergic, serotonergic, and dopaminergic circuits, although these associations are context-dependent and less well established [32,33,34]. These neuromodulatory systems regulate synaptic plasticity, attention, and emotional processing, and can in turn modulate neuroligin expression and function in a bidirectional manner [112,113,114]. Dopaminergic signaling can influence synaptic stabilization and plasticity through neuroligin-dependent mechanisms in reward-related circuits [115], while serotonergic signaling regulates developmental synaptogenesis by shaping neuroligin expression and synaptic maturation trajectories [116]. Cholinergic signaling similarly contributes to the regulation of synaptic development and plasticity across distributed neural circuits [117].

At the intracellular level, neuroligins activate multiple signaling cascades that convert extracellular adhesion events into long-term structural and functional changes. These include Rho GTPase-mediated regulation of cytoskeletal dynamics, which controls dendritic spine morphology and synaptic stability [118], as well as PI3K–Akt signaling pathways involved in synaptic growth [119]. Moreover, MAPK/ERK pathways link neuroligin activity to transcriptional programs underlying long-term synaptic plasticity [120]. Through the integration of these pathways, neuroligins couple synaptic adhesion to intracellular plasticity mechanisms, coordinating structural and functional adaptations across multiple temporal scales.

## 3. Neuroligins and Neuropathic Pain

### 3.1. Alteration of E/I Synaptic Balance

Peripheral nerve injury plays a key role in the reorganization of spinal dorsal horn circuits, in which the excitatory/inhibitory (E/I) synaptic balance shifts toward pathological hyperexcitability [121,122]. After nerve injury, inhibitory synaptic transmission in the dorsal horn is disrupted, and NLGN2 may be altered in inhibitory circuits. Under physiological conditions, NLGN2 is mainly localized at GABAergic and glycinergic synapses, where it recruits the scaffolding protein gephyrin, enabling stabilization of GABA_A_ and glycine receptors at the postsynaptic membrane [72,73,74]. This molecular organization is essential for maintaining inhibitory synaptic efficacy and chloride-dependent neuronal hyperpolarization.

In parallel, injury-induced changes in kinase activity, including enhanced GSK3β and ERK signaling, impair gephyrin clustering by altering its phosphorylation state and reducing its ability to oligomerize [123]. Increased proteolytic activity and ubiquitin–proteasome signaling further destabilize synaptic scaffolds, compromising inhibitory synapse integrity [124]. The overall consequence is a marked reduction in the density and functional efficacy of inhibitory postsynaptic receptors, leading to reduced inhibitory tone within spinal nociceptive circuits and loss of gating control over afferent sensory input.

On the other hand, excitatory glutamatergic transmission is relatively preserved or even enhanced, involving NLGN1-dependent mechanisms. NLGN1 is localized at excitatory synapses, where it interacts with PSD-95 and coordinates the assembly of AMPA- and NMDA receptor-containing complexes [31]. After nerve injury, synaptic remodeling promotes increased trafficking and stabilization of glutamate receptors, particularly GluA1-containing AMPA receptors and NR2B-enriched NMDA receptors [125,126]. This enhances synaptic efficacy and drives prolonged depolarization in response to afferent input. The resulting increase in Ca^2+^ influx through NMDA receptors activates intracellular signaling pathways, including CaMKII, MAPK/ERK, and PI3K–mTOR cascades, which further potentiate synaptic transmission [127]. These molecular events contribute to LTP-like changes in nociceptive neurons, reinforcing excitatory signaling within dorsal horn networks.

### 3.2. NMDA Receptor Potentiation via NLGN1 Pathways

NLGN1 plays a role in the functional potentiation of excitatory synapses in the spinal dorsal horn by organizing the postsynaptic glutamatergic machinery through its interaction with scaffold proteins of the postsynaptic density, particularly PSD-95 [23,31]. Following nerve injury, this molecular axis becomes increasingly relevant in shaping synaptic strength, as NLGN1 contributes to the stabilization and upregulation of NMDA receptor complexes, thus facilitating the synaptic hyperexcitability underlying neuropathic pain [128].

At the molecular level, NLGN1 acts as a trans-synaptic adhesion molecule that binds presynaptic neurexins while recruiting PSD-95 at the postsynaptic membrane [129]. PSD-95 serves as a central scaffolding hub that organizes glutamate receptor complexes, including NMDA and AMPA receptors, into highly efficient signaling microdomains [130]. Through this interaction, NLGN1 indirectly regulates NMDA receptor clustering at excitatory synapses, increasing receptor density at the postsynaptic membrane and enhancing the probability of channel activation during presynaptic glutamate release [128]. After nerve injury, this organization becomes exaggerated, promoting increased incorporation of NR2B-containing NMDA receptor subunits, which exhibit higher Ca^2+^ permeability and prolonged channel open times [131].

**Figure 3:**
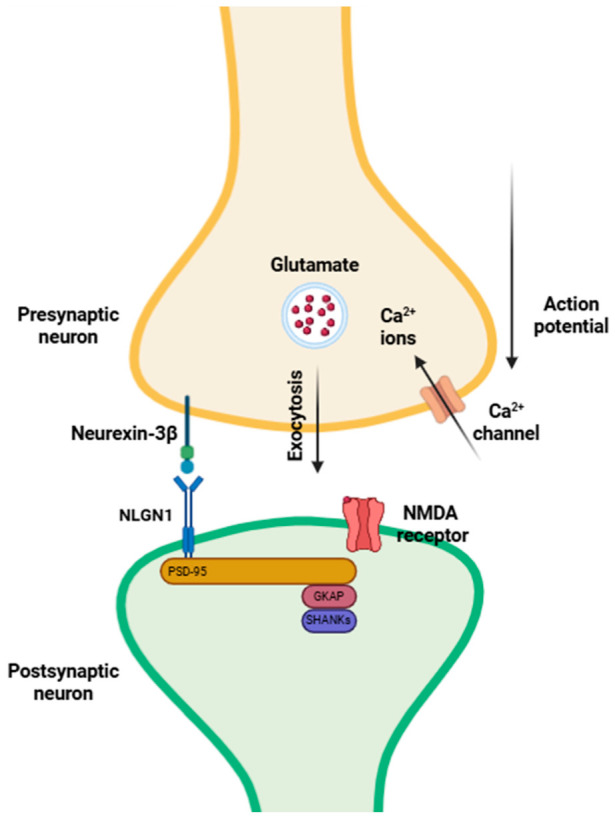
Organization of the glutamatergic synapse mediated by neurexin–neuroligin interactions. Schematic representation of an excitatory glutamatergic synapse showing the trans-synaptic interaction between presynaptic neurexin-3β and postsynaptic neuroligin-1. Glutamate released into the synaptic cleft (due to Ca^2+^ influx via activation of VGCCs) activates postsynaptic NMDA receptors, while intracellular scaffold proteins like PSD-95, GKAP, and SHANKs stabilize synaptic signaling complexes. Abbreviations: NLGN1 (neuroligin-1), Ca^2+^ (calcium ion), PSD-95 (postsynaptic density protein 95), GKAP (guanylate kinase-associated protein), and SHANK (SH3 and multiple ankyrin repeat domains protein).

This enhanced NMDA receptor clustering leads to increased postsynaptic Ca^2+^ influx during synaptic transmission. Elevated intracellular Ca^2+^ levels in dorsal horn neurons act as key second messengers driving synaptic plasticity processes associated with central sensitization [132]. In particular, Ca^2+^ entry through NMDA receptors activates calmodulin, which subsequently triggers CaMKII. Once activated, CaMKII undergoes autophosphorylation, allowing it to remain persistently active even after Ca^2+^ levels decline, thus converting transient synaptic activity into long-lasting changes in synaptic strength (Figure 3) [133,134].

Downstream of CaMKII activation, multiple phosphorylation cascades are engaged that further potentiate excitatory synaptic transmission. These include phosphorylation of AMPA receptor subunits, predominantly GluA1, which increases receptor conductance and promotes synaptic insertion [135]. Simultaneously, activation of MAPK/ERK signaling pathways induces transcriptional changes via CREB phosphorylation, enhancing the expression of genes involved in synaptic growth and maintenance [136]. Moreover, PI3K–mTOR signaling contributes to local dendritic protein synthesis, further stabilizing potentiated synapses [137].

The convergence of these mechanisms results in sustained amplification of NMDA receptor-mediated signaling within dorsal horn neurons. NLGN1-driven organization of PSD-95 complexes not only increases receptor availability but also ensures efficient coupling between receptor activation and intracellular signaling pathways [129]. This molecular coupling is critical for the transition from acute nociceptive processing to persistent synaptic sensitization.

### 3.3. Inhibitory Synapse Destabilization by NLGN2

**Figure 4:**
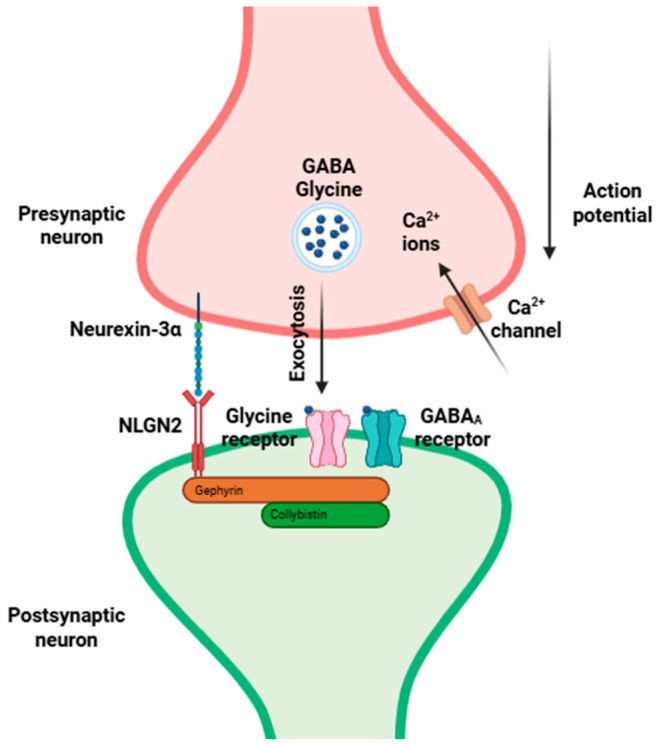
Schematic illustration of an inhibitory GABAergic/glycinergic synapse highlighting the trans-synaptic interaction between presynaptic neurexin-3α and postsynaptic NLGN2. Neurotransmitter release of GABA/glycine into the synaptic cleft, triggered by Ca^2+^ influx via VGCCs, leads to activation of postsynaptic GABA_A_ and glycine receptors, while gephyrin and collybistin act as scaffold proteins that stabilize inhibitory postsynaptic receptor complexes. Abbreviations: Ca^2+^ (calcium ion), GABA (gamma-aminobutyric acid), GABA_A_ (gamma-aminobutyric acid type A receptor), and NLGN2 (neuroligin-2).

NLGN2 is a central molecular organizer of inhibitory synapses in the spinal dorsal horn, where it ensures the assembly, stabilization, and functional maintenance of GABAergic and glycinergic transmission [72,73,74]. Under physiological conditions, NLGN2 is selectively enriched at inhibitory postsynaptic densities, where it forms a trans-synaptic adhesion complex with presynaptic neurexins [138]. This interaction is not solely structural but also instructive, as it promotes the recruitment of intracellular scaffolding proteins, most notably gephyrin, which serves as the principal platform for the assembly of GABA_A_ and glycine receptors within postsynaptic nanodomains (Figure 4) [139]. This architecture is essential for generating fast, reliable inhibitory postsynaptic currents that tightly regulate nociceptive signal propagation within spinal circuits [140].

Following peripheral nerve injury, a consistent molecular signature is the reduction in NLGN2 expression and/or membrane stability in dorsal horn neurons [141]. This downregulation may result from inflammatory transcriptional repression, activity-dependent synaptic remodeling, and disruptions in proteostasis within injured spinal networks [142,143,144]. As NLGN2 levels decline, the stability of the NLGN2–neurexin adhesion complex is compromised, weakening the extracellular anchoring of inhibitory synapses [93]. More critically, gephyrin recruitment becomes less efficient, impairing its ability to oligomerize into higher-order scaffolds. Because gephyrin clustering is crucial for the capture and stabilization of GABA_A_ and glycine receptors at synapses, its destabilization affects postsynaptic receptor organization [145,146].

This process results in the collapse of inhibitory receptor microdomains. GABA_A_ receptors, which normally organize into dense nanoclustered arrays aligned with presynaptic release sites, become dispersed within the membrane or exhibit increased lateral diffusion away from synaptic domains [147]. Glycine receptors also lose their postsynaptic sequestration [148]. This reduction in receptor clustering significantly decreases the efficacy of inhibitory synaptic transmission, as fewer receptors are available at active sites during vesicular neurotransmitter release [149]. As a result, inhibitory postsynaptic currents become smaller in amplitude, less synchronized, and more prone to failure during high-frequency afferent activity [140].

Functionally, the weakening of inhibitory synapses produces a marked reduction in synaptic gain control within dorsal horn networks. Under normal conditions, GABAergic and glycinergic interneurons regulate the excitability of projection neurons by limiting temporal summation of excitatory inputs and filtering nociceptive afferent activity [150]. However, after NLGN2 loss, this inhibitory gating becomes ineffective, allowing subthreshold excitatory inputs to more readily trigger action potential firing [93]. This disinhibition is particularly pronounced in lamina II circuits, where sensory integration and nociceptive modulation critically depend on a finely balanced inhibitory tone [142].

This synaptic destabilization is further compounded by alterations in inhibitory synaptic architecture, mainly involving downregulation of NLGN2, which contributes to disrupted clustering and reduced stabilization of inhibitory postsynaptic receptors [151]. Under neuropathic pain conditions, NLGN2 loss occurs concomitantly with reduced stability of inhibitory synapses, weakening the structural scaffold required for efficient GABAergic transmission [152]. This destabilization compromises the organization of receptor complexes at the postsynaptic membrane, thereby reducing the overall efficacy of inhibitory signaling [153,154].

Finally, the combination of decreased receptor clustering and impaired Cl^−^ extrusion produces a synergistic collapse of inhibitory control. Although presynaptic inhibitory interneurons remain active, their postsynaptic impact is severely attenuated or functionally reversed. This results in a state of network disinhibition in which excitatory synaptic inputs from primary afferents or local excitatory interneurons are no longer counterbalanced [155].

### 3.4. Synaptic Plasticity and Central Sensitization

Neuroligins play a key role in activity-dependent synaptic plasticity within the spinal dorsal horn, where their differential regulation after nerve injury contributes to the structural and functional remodeling underlying central sensitization [22]. Rather than acting solely as static adhesion molecules, neuroligins dynamically regulate synapse formation, stabilization, and elimination in response to changes in neuronal activity [156]. In neuropathic pain states, this results in a coordinated reorganization of excitatory and inhibitory synaptic architecture, favoring persistent hyperexcitability [157].

Following peripheral nerve injury, excitatory neurons in the dorsal horn show a significant increase in dendritic spine density, a defining feature of structural synaptic plasticity [158]. This process is associated with NLGN1, whereby increased NLGN1 expression or stabilization enhances the recruitment of synaptic components required for spine growth, including regulators of the actin cytoskeleton and receptor trafficking machinery. At the molecular level, activity-dependent Ca^2+^ influx, primarily through NMDA receptors, activates signaling pathways such as CaMKII, Rac1, and Rho GTPases, which drive actin polymerization and spine enlargement [159]. As a consequence, dendritic spines increase in number and undergo morphological maturation into mushroom-shaped structures characterized by prominent postsynaptic densities, supporting stronger and more stable excitatory synaptic transmission [160].

Concomitant with this increase in excitatory connectivity, there is a progressive loss of inhibitory synaptic contacts mediated by NLGN2 dysfunction. As NLGN2 expression declines after nerve injury, the stability of inhibitory synapses is compromised, leading to the elimination of GABAergic and glycinergic terminals [22]. This synaptic pruning is structural as well as functional, involving disassembly of gephyrin scaffolds, reduced anchoring of inhibitory receptors, and eventual loss of synaptic contact points between inhibitory interneurons and their target cells [161]. The result is a reduction in the number of functional inhibitory synapses onto dorsal horn neurons, further shifting the balance toward excitation [150]. Importantly, this loss of inhibitory connectivity is spatially selective, typically affecting perisomatic and proximal dendritic regions that are fundamental for controlling neuronal output [142].

In parallel, excitatory synapses undergo molecular strengthening through enhanced trafficking and insertion of AMPA receptors, particularly those containing the GluA1 subunit [162]. NLGN1-dependent stabilization of postsynaptic densities supports AMPA receptor recruitment to synaptic sites, a process tightly regulated by phosphorylation events downstream of Ca^2+^ signaling [163]. Activation of CaMKII and PKA promotes phosphorylation of the GluA1 subunit at specific serine residues (e.g., Ser831 and Ser845), increasing channel conductance and driving receptor insertion into the postsynaptic membrane [164]. Moreover, interactions with scaffolding proteins, including PSD-95 and auxiliary subunits such as TARPs, stabilize these receptors at synaptic sites, reducing endocytosis and enhancing synaptic efficacy [165].

The combined increase in dendritic spine density, loss of inhibitory synaptic contacts, and enhanced AMPA receptor-mediated transmission results in a profound reconfiguration of synaptic networks within the dorsal horn. These changes are not transient but become stabilized through activity-dependent gene expression programs involving transcription factors such as CREB, which promote synthesis of synaptic proteins that consolidate LTP-like states [166]. This long-lasting synaptic strengthening, together with reduced inhibitory control, forms the cellular basis of central sensitization.

### 3.5. Neuroimmune Interactions

Emerging evidence indicates that neuroligins are not isolated determinants of synaptic architecture but are embedded within neuroimmune signaling networks activated after peripheral nerve injury. In neuropathic pain states, the spinal dorsal horn undergoes a coordinated neuron–glia response in which microglia act as primary sensors of injury-induced signals [167]. These include ATP released from damaged afferents, GM-CSF, CX3CL1, and other damage-associated molecular patterns (DAMPs) [168]. Through receptors such as P2X4, TLR4, and CX3CR1, microglia transition into a reactive phenotype characterized by transcriptional reprogramming and robust secretion of neuromodulatory and pro-inflammatory factors, notably BDNF, TNF-α, and IL-1β [169,170]. These mediators do not simply produce transient changes in neuronal excitability; rather, they induce long-lasting modifications in synaptic organization, in part by regulating the expression and function of synaptic adhesion molecules, including neuroligins [171].

At the molecular level, cytokine signaling exerts both transcriptional and post-translational control over synaptic proteins. TNF-α, acting primarily through TNFR1, activates intracellular cascades including NF-κB, p38 MAPK, and JNK pathways [172,173]. These signaling routes modulate gene expression programs affecting synaptic components such as NLGN1, PSD-95, and glutamate receptor subunits [129,174]. Concurrently, TNF-α can rapidly influence synaptic composition through non-genomic mechanisms, including promotion of AMPA receptor exocytosis and inhibition of receptor endocytosis [175]. Within this framework, NLGN1 serves as a structural and functional hub, as its interaction with PSD-95 stabilizes postsynaptic densities and provides anchoring sites for AMPA receptors [34]. Under inflammatory conditions, TNF-α evokes the accumulation of GluA1-containing AMPA receptors at synaptic membranes, a process facilitated by NLGN1-dependent organization of the postsynaptic scaffold [176]. This leads to increased synaptic conductance, enhanced responsiveness to glutamate release, and sustained potentiation of excitatory transmission [177]. Importantly, this mechanism links neuroinflammatory signaling directly to structural synaptic reinforcement, coupling immune activation to long-term plasticity.

Beyond its effects on AMPA receptors, TNF-α indirectly strengthens NMDA receptor function by increasing receptor phosphorylation and promoting the incorporation of NR2B-containing subunits, which exhibit higher Ca^2+^ permeability [178]. The resulting elevation in intracellular Ca^2+^ activates downstream kinases including CaMKII and ERK, reinforcing activity-dependent synaptic enhancement [179]. These pathways overlap with NLGN1-mediated synaptic stabilization, creating a feedforward loop in which inflammation enhances excitatory synapse maturation and persistence [180]. Over time, this contributes to the consolidation of LTP-like states within nociceptive circuits, a hallmark of central sensitization [181].

In parallel, BDNF released from activated microglia exerts a modulatory influence on inhibitory synaptic function, primarily through activation of the TrkB receptor. One key downstream consequence of BDNF–TrkB signaling is downregulation of the K^+^–Cl^−^ cotransporter KCC2 [182]. Reduced KCC2 expression leads to intracellular Cl^−^ accumulation and a depolarizing shift in the chloride equilibrium potential, resulting in reduced hyperpolarizing efficacy or, in some cases, paradoxical depolarizing responses upon activation of GABA_A_ or glycine receptors. Although mechanistically distinct from NLGN2 dysfunction, this pathway converges with it through complementary processes: NLGN2 loss destabilizes inhibitory synaptic scaffolds and reduces receptor clustering, whereas BDNF-induced Cl^−^ dysregulation compromises the ionic gradient required for inhibitory signaling [183]. Together, these processes lead to a progressive breakdown of inhibitory control at both structural and electrochemical levels.

IL-1β further contributes to this neuroinflammatory modulation by enhancing excitatory synaptic transmission through multiple mechanisms. Binding to its receptor (IL-1R1), IL-1β activates intracellular pathways including p38 MAPK and Src family kinases, which increase NMDA receptor phosphorylation and channel activity [184,185]. This potentiates Ca^2+^ influx and amplifies downstream signaling cascades associated with synaptic plasticity [186]. Additionally, IL-1β can influence gene expression programs regulating synaptic proteins, potentially affecting neuroligin expression and turnover [187]. Through these actions, IL-1β reinforces excitatory signaling and indirectly contributes to NLGN1-dependent synaptic strengthening.

Beyond direct cytokine effects, astrocytes also participate in this neuroimmune–synaptic interface by modulating extracellular glutamate levels. Reactive astrocytes downregulate glutamate transporters such as GLT-1 (EAAT2), leading to prolonged glutamate presence in the synaptic cleft and enhanced activation of postsynaptic receptors [188]. This excess glutamate further engages NLGN1-organized synapses, promoting sustained activation of NMDA and AMPA receptors and reinforcing excitatory synaptic plasticity [189]. Thus, astrocytic dysfunction complements microglial signaling in amplifying synaptic imbalance.

Importantly, the interaction between neuroinflammation and neuroligins is self-reinforcing. Strengthened excitatory signaling mediated by NLGN1-dependent processes elevates neuronal activity and neurotransmitter release, which further stimulates microglial activation and cytokine production. Conversely, pro-inflammatory mediators continuously reshape synaptic adhesion and receptor organization, stabilizing maladaptive circuit configurations. This creates a persistent feedforward loop in which synaptic plasticity and neuroinflammation mutually sustain each other over time.

## 4. Therapeutic Implications

### 4.1. Neuroligins as Emerging Targets for RNAi-Based Therapeutic Intervention

The growing evidence implicating neuroligins in synaptic dysfunction and maladaptive plasticity has positioned these molecules as promising therapeutic targets for neuropathic pain. Because neuroligins critically regulate excitatory–inhibitory neurotransmission, pharmacological modulation of their activity may help restore synaptic homeostasis and attenuate persistent nociceptive signaling. In neuropathic pain, alterations in neuroligin expression and function contribute to central sensitization, aberrant neuronal excitability, and impaired inhibitory control within pain-processing circuits. Consequently, interventions aimed at modulating neuroligin-dependent synaptic organization could provide disease-modifying benefits rather than solely symptomatic relief.

RNA-based therapeutic strategies represent a particularly promising approach. RNA interference (RNAi) enables selective modulation of specific neuroligin isoforms implicated in pain pathways, providing a targeted strategy for regulating aberrant synaptic signaling associated with chronic pain states (Table 2) [190]. Furthermore, the molecular specificity of RNAi minimizes off-target effects, highlighting its potential as a precision therapeutic platform for the treatment of neuropathic pain disorders [191].

Despite their therapeutic promise, neuroligin-targeted interventions remain limited by substantial biological, technical, and translational challenges that must be addressed before clinical application becomes feasible. One of the principal limitations lies in the highly specific and heterogeneous expression patterns of neuroligins across neuronal populations and brain regions. Each neuroligin isoform exhibits distinct localization and functional roles at excitatory and inhibitory synapses, meaning that even subtle, non-selective modulation may disrupt synaptic organization and overall network stability [193].

This issue of synaptic specificity is particularly relevant in neuropathic pain, where nociceptive circuits are closely interconnected with systems involved in cognition, emotional regulation, sensory integration, and motor control [194]. As a result, therapeutic strategies must achieve precise spatial and cell-type specificity to avoid unintended disruption of physiological neurotransmission. Even minor alterations in excitatory–inhibitory balance may lead to adverse neurological effects, including cognitive impairment, emotional dysregulation, and a range of neuropsychiatric symptoms [195].

In addition, neuroligins play key roles in neurodevelopment and synaptic maintenance throughout the CNS, and their dysregulation has been implicated in numerous neurodevelopmental and psychiatric disorders, including autism spectrum disorder (ASD), schizophrenia, intellectual disability, and epilepsy [102,196]. Accordingly, increased modulation of neuroligin function raises concerns regarding off-target effects and unintended disturbances in synaptic plasticity, circuit maturation, and higher-order brain functions.

Further complexity arises from the intrinsic heterogeneity of neuropathic pain itself, which varies widely depending on etiology, anatomical distribution, inflammatory status, genetic background, and disease chronicity. This variability suggests that neuroligin-associated mechanisms may differ across patient populations, reducing the likelihood that a single therapeutic strategy will be broadly effective.

Finally, although preclinical studies have shown encouraging results in terms of synaptic modulation and pain attenuation, the translational gap between animal models and human neuropathic pain remains a major obstacle. Rodent models, in particular, fail to fully capture the multifactorial and subjective nature of chronic pain in humans [197]. As a result, extensive preclinical validation and carefully designed clinical studies will be required to optimize targeting strategies, establish therapeutic windows, and ensure long-term safety and efficacy before neuroligin-based interventions can be considered for routine clinical use.

### 4.2. Future Therapeutic Strategies

Future therapeutic strategies targeting neuroligins may evolve toward increasingly personalized, multimodal, and molecularly refined interventions. However, despite substantial advances in the understanding of synaptic dysfunction in neuropathic pain, the clinical translation of neuroligin-targeted therapies remains at an early stage. Many of the approaches discussed below are still largely experimental, and their long-term efficacy, safety, and feasibility in clinical settings remain uncertain. Further research is required to determine whether emerging technologies can achieve precise modulation of maladaptive neural circuits while preserving physiological synaptic function.

One potential direction involves personalized medicine approaches. Emerging evidence suggests that interindividual variability in neuroligin expression, genetic polymorphisms, and synaptic architecture may contribute to differences in susceptibility to neuropathic pain and treatment responsiveness. Comprehensive molecular profiling of patients, including genomic, transcriptomic, proteomic, and epigenomic analyses, has been proposed as a strategy to identify neuroligin-related pathological signatures [198]. Nevertheless, the clinical utility, reproducibility, scalability, and cost-effectiveness of such approaches remain to be established. If successfully validated, these strategies may facilitate patient stratification and support the development of more individualized therapeutic interventions.

Precision medicine approaches may also benefit from biomarker-guided treatment selection. Neuroligin isoform expression patterns, pro-inflammatory mediators, and synaptic protein networks have been proposed as potential biomarkers of disease progression and therapeutic response [199,200]. However, their predictive utility remains uncertain and requires further validation in large, well-characterized patient cohorts. The integration of artificial intelligence and machine-learning methods with large-scale omics datasets may assist in identifying relevant molecular targets and improving therapeutic decision-making. Nevertheless, limitations related to dataset quality, heterogeneity, and availability, together with challenges in data standardization, interpretability, and clinical implementation, may restrict the generalizability of these approaches [201,202].

In parallel, emerging biomolecular approaches are expanding the range of potential therapeutic options. Refined RNA-based technologies, including siRNA, miRNA modulation, antisense oligonucleotides (ASOs), and mRNA-based delivery systems, offer opportunities for selective regulation of neuroligin expression at the post-transcriptional level [203]. Similarly, epigenetic interventions targeting histone modifications, DNA methylation, or chromatin-remodeling pathways may provide novel strategies for modulating maladaptive synaptic plasticity associated with chronic pain [204]. However, important challenges remain regarding delivery efficiency, target specificity, durability of therapeutic effects, and potential off-target consequences. Furthermore, long-term safety profiles remain largely unknown.

Another area of interest involves the development of engineered biologics designed to selectively modulate pathological neuroligin–neurexin interactions while minimizing disruption of physiological synaptic connectivity. Although conceptually attractive, achieving sufficient molecular specificity without affecting normal synaptic function remains challenging. Nanotechnology-based delivery systems, such as lipid nanoparticles and exosome-mediated platforms, may enhance therapeutic targeting and blood–brain barrier (BBB) penetration while potentially reducing systemic adverse effects [205]. Nevertheless, issues related to biodistribution, immunogenicity, manufacturing scalability, and regulatory approval remain incompletely resolved. Substantial technical and translational barriers must therefore be addressed before these approaches can be considered clinically applicable.

Likewise, optogenetic and chemogenetic technologies offer powerful experimental tools for activity-dependent modulation of pain circuits with high temporal and spatial precision. While these approaches have shown promising results in preclinical models, their translation to human applications faces considerable biological, technical, and ethical challenges. Consequently, their long-term feasibility, safety, and therapeutic utility in patients remain uncertain [206]. In combination with neuroligin-targeted molecular interventions, these technologies may provide valuable insights into the mechanisms underlying pathological nociceptive signaling, although their future clinical role remains speculative.

Finally, stem-cell-based regenerative therapies have been suggested as a key strategy for restoring dysfunctional neural circuits involved in chronic pain. Neural stem cells or induced pluripotent stem cells (iPSCs) engineered to express specific synaptic regulatory molecules may contribute to repairing damaged neural networks and re-establishing inhibitory signaling [207]. However, considerable challenges related to cell survival, differentiation, integration into existing neural circuits, tumorigenic risk, long-term safety, and regulatory approval must be overcome before such approaches can be considered viable therapeutic options.

Overall, although neuroligin-targeted strategies represent a potentially valuable area of investigation, most approaches remain at the preclinical stage, and several fundamental questions regarding target specificity, long-term safety, and clinical efficacy remain unanswered. Future studies should prioritize rigorous validation in clinically relevant models, the identification of reliable biomarkers, and well-designed translational investigations. Addressing these challenges will be crucial before neuroligin-based interventions can be realistically considered for routine clinical application in neuropathic pain management.

## 5. Conclusions

The evidence presented in this review underscores the role of neuroligins as fundamental organizers of synaptic structure and function within nociceptive pathways. The accumulated findings support the view that neuroligins are not merely structural adhesion molecules; instead, they act as regulators of synaptic plasticity that influence the balance between excitatory and inhibitory transmission. This balance is essential for shaping neuronal circuit excitability, and its disruption appears to be a central feature in the transition from physiological pain processing to chronic neuropathic pain states.

In neuropathic pain conditions, altered neuroligin expression or function contributes to maladaptive synaptic remodeling, leading to persistent hyperexcitability within spinal and supraspinal pain pathways. This synaptic dysregulation enhances nociceptive signal transmission and reduces inhibitory control, thereby enabling pain amplification and maintenance. In this context, neuroligins emerge as key molecular elements linking synaptic architecture with functional pain modulation, positioning them as potential drivers in the pathophysiology of chronic pain. From a broader perspective, neuroligins represent promising therapeutic targets due to their central role in synaptic organization and plasticity. Modulating neuroligin-mediated interactions could offer a strategy to restore synaptic balance and reduce pathological pain signaling.

However, several important gaps remain in the field. In particular, potential sex-dependent differences in neuroligin expression and function within pain pathways remain poorly characterized. Neuroimmune studies have provided evidence suggesting that sex hormones and sex-specific transcriptional programs may influence synaptic organization and plasticity, raising the possibility that neuroligin-mediated mechanisms could also exhibit sexually dimorphic patterns. Such differences may contribute to variability in susceptibility to neuropathic pain, as well as differential responses to analgesic interventions, yet this aspect has not been systematically investigated.

In addition, the isoform-specific roles of neuroligins in different pain modalities are not yet fully understood. Although NLGN1 is generally associated with excitatory synapses and NLGN2 with inhibitory synapses, their exact contributions within nociceptive circuits remain unclear. It is not yet established whether these isoforms act in a compensatory, redundant, or opposing manner across stages of neuropathic pain development, which limits the mechanistic interpretation of current findings. Moreover, the cell-type-specific contributions of neuroligins in neurons and glial cells, as well as their stage-dependent roles in neuropathic pain, remain to be further characterized. These unresolved questions underscore the complexity of neuroligin signaling in pain circuits and the need for more refined experimental approaches.

An additional key unresolved issue is whether neuroligin alterations represent primary causal drivers of maladaptive synaptic remodeling in neuropathic pain or whether they arise as secondary consequences of ongoing pathological activity and circuit reorganization. Although several experimental models suggest that neuroligin dysregulation contributes to altered synaptic strength and excitability, other studies propose that these changes may instead represent activity-dependent adaptations following chronic nociceptive input. This bidirectional ambiguity hinders causal inference and highlights the importance of longitudinal studies and experimental manipulation approaches (e.g., conditional knockouts or temporally controlled overexpression models) in distinguishing causality from downstream consequences.

Future research should prioritize validation of neuroligin-related mechanisms in clinical settings through well-designed translational and clinical studies. Establishing whether neuroligins can serve as reliable biomarkers or therapeutic targets in human neuropathic pain will be a key step forward. Moreover, integrating emerging high-throughput technologies such as transcriptomics, proteomics, and metabolomics with advanced imaging techniques will be essential to achieve a more comprehensive understanding of neuroligin-mediated synaptic alterations. These approaches will allow researchers to capture both molecular and functional changes across spatial and temporal scales.

Overall, neuroligins represent a highly promising yet still insufficiently characterized component of pain neurobiology. Their involvement in synaptic plasticity and nociceptive transmission provides a strong framework for future research aimed at developing more effective, mechanism-based therapies for neuropathic pain. Addressing the aforementioned gaps will be essential for translating basic synaptic findings into clinically meaningful interventions.

## Figures and Tables

**Table 1:** Structural and functional overview of human neuroligin family members: Abbreviations: NLGN1 (neuroligin-1); bp (base pairs); Da (Dalton); AMPA (α-amino-3-hydroxy-5-methyl-4-isoxazolepropionic acid); NLGN2 (Neuroligin-2); GABA_A_ (gamma-aminobutyric acid type A receptor); NLGN3 (neuroligin-3); PSD-95 (postsynaptic density protein 95); NLGN4X (Neuroligin-4X); ASDs (autism spectrum disorders); and NLGN4Y (neuroligin-4Y).

Member	Gene Size and Chromosomal Localization (Human)	Protein Size (Human)	CNS Expression	Functional Actions	References
NLGN1	898,421 bp (3q26.31)	863 amino acids (96.37 kDa)	Prefrontal cortex Hippocampus Spinal cord	Interacts with β-neurexins to promote excitatory synapse formation, maturation, and AMPA receptor clustering Triggers presynaptic differentiation in vitro	[23,31,34,69,70,71]
NLGN2	15,208 bp (17p13.1)	835 amino acids (90.82 kDa)	Midbrain Lateral septum Prefrontal cortex Spinal cord	Mediates inhibitory synapse specification via neurexin binding and recruitment of GABA_A_ receptor complexes Balances excitatory–inhibitory signaling ratios	[22,70,72,73,74,75,76]
NLGN3	32,022 bp (Xq13.1)	848 amino acids (93.9 kDa)	Cerebellum Hippocampus Spinal cord	Circuit-specific organizer of both excitatory and inhibitory synapses via splice isoform-dependent neurexin interactions Stabilizes PSD-95 and enhances synaptic transmission efficiency	[77,78,79,80,81]
NLGN4X	388,231 bp (Xp22.32-p22.31)	816 amino acids (91.92 kDa)	Prefrontal cortex Hippocampus Spinal cord	Postsynaptic ligand for β-neurexins primarily at excitatory synapses in the cerebral cortex Implicated in synapse remodeling, with mutations linked to ASDs	[82,83,84,85,86,87]
NLGN4Y	323,082 bp (Yq11.221)	816 amino acids (92.02 kDa)	Prefrontal cortex Spinal cord	Potential role in male-specific synaptic adhesion Interacts with neurexins for synapse maturation	[88,89,90]

**Table 2:** Preclinical effects of siRNA-mediated silencing of neuroligin-1 and neuroligin-2 pain behaviors in various rodent models of neuropathic pain. Abbreviations: siRNA (small interfering RNA), NLGN1 (neuroligin-1), GluA1 (glutamate receptor AMPA subtype 1), PSD-95 (postsynaptic density protein 95), SNL (spinal nerve ligation), NLGN2 (neuroligin-2), E/I (excitatory/inhibitory balance), NR2B (NMDA receptor subunit 2B), and pNR2B (phosphorylated NMDA receptor subunit 2B).

RNA Strategy	Model	Key Findings	References
siRNA against NLGN1	Rat postoperative pain	In a rat model of postoperative pain induced by plantar incision, spinal NLGN1 expression was significantly increased in the ipsilateral dorsal horn at 3 h and 1 day after surgery, together with elevated GluA1 levels, whereas GluA2 and PSD-95 expression remained unchanged. Coimmunoprecipitation analyses revealed enhanced NLGN1/PSD-95 interaction at 3 h post-incision. Intrathecal administration of NLGN1-targeting siRNA prevented GluA1 upregulation, reduced NLGN1/PSD-95 interaction, and significantly attenuated postoperative pain behaviors. These findings suggest that spinal NLGN1 contributes to postoperative hypersensitivity by promoting excitatory synaptic transmission through PSD-95-dependent GluA1 recruitment.	[34]
siRNA against NLGN2	Rat SNL	In the SNL model of neuropathic pain, the E/I balance in the spinal dorsal horn shifts toward hyperexcitability. Unexpectedly, and in contrast to its canonical inhibitory function, NLGN2 showed a paradoxical role in the SNL condition. NLGN2 expression was significantly upregulated. In control rats, intrathecal NLGN2 siRNA increased mechanical sensitivity, confirming its inhibitory role; however, in SNL rats, NLGN2 knockdown produced antinociceptive effects, indicating a pathological pronociceptive shift. Mechanistically, NLGN2 overexpression was associated with increased PSD-95 expression, colocalization, and interaction, while gephyrin levels were unchanged.	[122]
siRNA against NLGN1	Rat SNL	In the SNL model of neuropathic pain, mechanical allodynia was associated with a significant increase in pNR2B expression in the spinal dorsal horn. Although NLGN1 and PSD-95 total expression remained unchanged, SNL markedly enhanced NLGN1/PSD-95, pNR2B/PSD-95, and NLGN1/NR2B interactions at day 7 post-injury Immunofluorescence analyses further confirmed increased neuronal colocalization of NLGN1, PSD-95, and pNR2B in the ipsilateral dorsal horn. Blocking NLGN1 signaling with NLGN1-targeting siRNA prevented these molecular interactions and reduced neuropathic pain behaviors. These findings indicate that NLGN1 contributes to neuropathic pain by facilitating PSD-95-dependent NR2B phosphorylation and excitatory synaptic signaling in the spinal dorsal horn.	[192]

## Data Availability

No new data were created or analyzed in this study. Data sharing is not applicable to this article.

## Abbreviations

The following abbreviations are used in this manuscript:

AI	Artificial intelligence
AMPA	α-amino-3-hydroxy-5-methyl-4-isoxazolepropionic acid receptor
ASD	Autism spectrum disorder
ASO	Antisense oligonucleotide
ATP	Adenosine triphosphate
BBB	Blood–brain barrier
bp	Base pairs
Ca^2+^	Calcium ion
CaMKII	Calcium/calmodulin-dependent protein kinase II
CCL2	C-C motif chemokine ligand 2
CCL4	C-C motif chemokine ligand 4
ChE-like	Cholinesterase-like
CIPN	Chemotherapy-induced peripheral neuropathy
Cl^−^	Chloride ion
CNS	Central nervous system
CREB	cAMP response element-binding protein
C-terminal	Carboxy-terminal
CX3CR1	C-X3-C motif chemokine receptor 1
Da	Dalton
DAMP	Damage-associated molecular pattern
DNA	Deoxyribonucleic acid
DPN	Diabetic peripheral neuropathy
E/I	Excitatory/inhibitory balance
EAAT2	Excitatory amino acid transporter 2
ERK	Extracellular signal-regulated kinase
GABA	Gamma-aminobutyric acid
GABA_A_	Gamma-aminobutyric acid type A receptor
GKAP	Guanylate kinase-associated protein
GLT-1	Glutamate transporter-1 (also known as EAAT2)
Glu	Glutamate
GluA1	Glutamate receptor AMPA subtype 1
GluA2	Glutamate receptor AMPA subtype 2
GM-CSF	Granulocyte-macrophage colony-stimulating factor
GSK3β	Glycogen synthase kinase 3 beta
GTPase	Guanosine triphosphate phosphatase
His	Histidine
IGF-1	Insulin-like growth factor 1
IL-1R1	Interleukin-1 receptor type 1
IL-1β	Interleukin-1 beta
IL-8	Interleukin 8
iPSC	Induced pluripotent stem cell
JNK	c-Jun N-terminal kinase
K^+^	Potassium ion
KCC2	Potassium-chloride cotransporter 2
LNS	Laminin–neurexin–sex hormone-binding globulin domains
LTD	Long-term depression
LTP	Long-term potentiation
MAGUK	Membrane-associated guanylate kinase
MAPK	Mitogen-activated protein kinase
MAPK/ERK	Mitogen-activated protein kinase/extracellular signal-regulated kinase
Mg^2+^	Magnesium ion
mRNA	Messenger RNA
miRNA	MicroRNA
MMP-13	Matrix metalloproteinase 13
MMP-3	Matrix metalloproteinase 3
MS	Multiple sclerosis
mTOR	Mechanistic target of rapamycin
NF-κB	Nuclear factor kappa-light-chain-enhancer of activated B cells
NLGN1	Neuroligin-1
NLGN2	Neuroligin-2
NLGN3	Neuroligin-3
NLGN4	Neuroligin-4
NLGN4X	Neuroligin-4X
NLGN4Y	Neuroligin-4Y
NMDA	N-methyl-D-aspartate receptor
NR2B	NMDA receptor subunit 2B
P2X4	Purinergic receptor P2X4
p38 MAPK	p38 mitogen-activated protein kinase
PI3K-Akt	Phosphoinositide 3-kinase
PKA	Protein kinase A
PKB/Akt	Protein kinase B
pNR2B	Phosphorylated NMDA receptor subunit 2B
PSD-95	Postsynaptic Density protein 95
Rac1	Ras-related C3 botulinum toxin substrate 1 (a small GTPase)
Rho GTPase	Ras homolog gene family/guanosine triphosphatase protein
RNA	Ribonucleic acid
RNAi	RNA interference
SAP102	Synapse-associated protein 102
SASP	Senescence-associated secretory phenotype
Ser	Serine
SHANK	SH3 and multiple ankyrin repeat domains protein
siRNA	Small interfering RNA
SNL	Spinal nerve ligation
TARP	Transmembrane AMPA receptor regulatory protein
TGF-β	Transforming growth factor beta
TLR4	Toll-like receptor 4
TNFR1	Tumor necrosis factor receptor 1
TNF-α	Tumor necrosis factor alpha
TrkB	Tropomyosin receptor kinase B
VGCC	Voltage-gated calcium channel
